# Supplementary material for: The causal relationship between the human gut microbiota and pyogenic arthritis: a Mendelian randomization study
Source: Front Cell Infect Microbiol. 2024 Nov 26;14:1452480. doi: 10.3389/fcimb.2024.1452480 (PMC11629706; doi:10.3389/fcimb.2024.1452480)
Supplement: Supplementary file 1 [file DataSheet1.zip › mendelian test/ebi-a-GCST90027824.csv_leave_one_out.pdf]

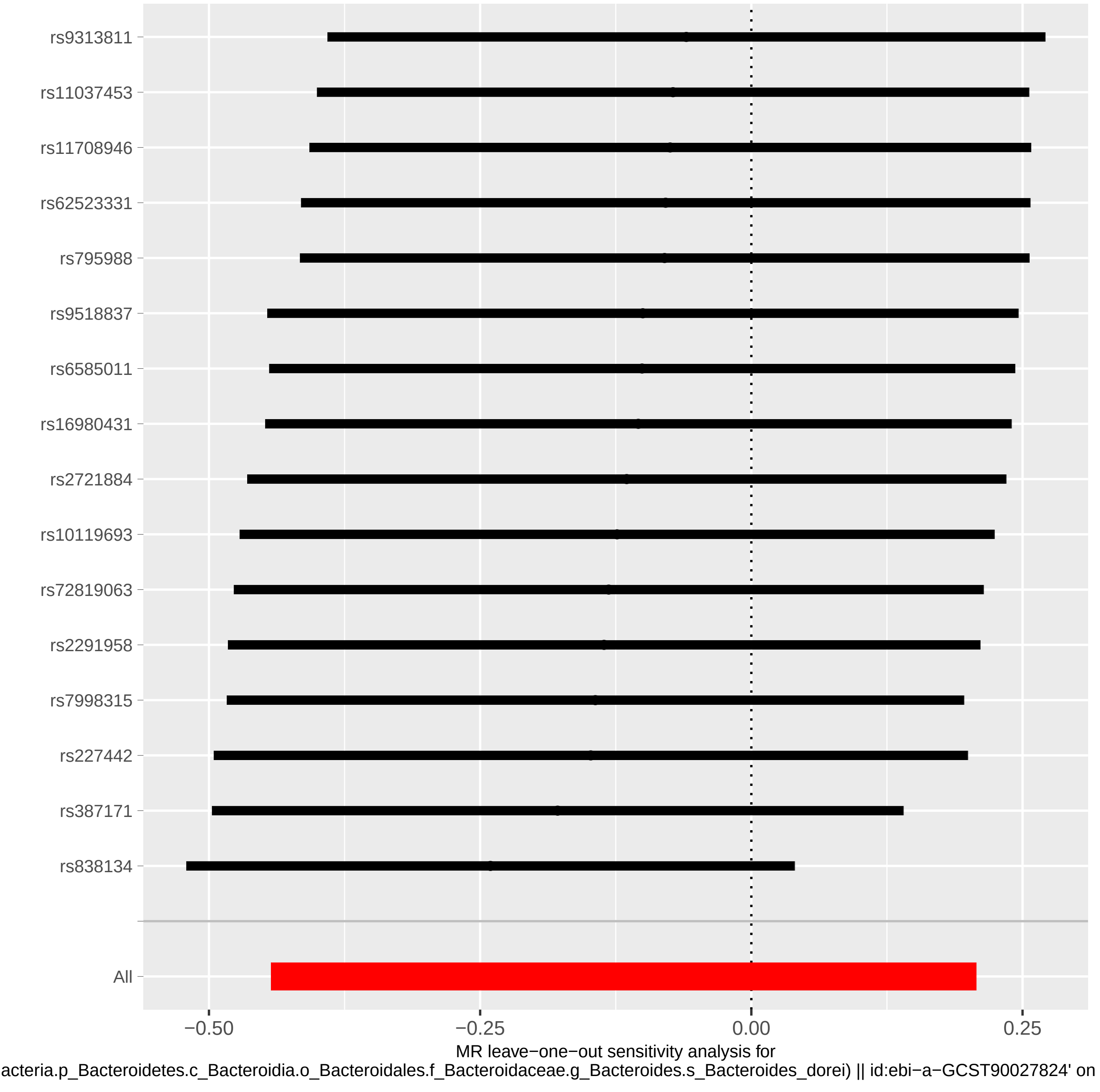

rs9313811
rs11037453
rs11708946
rs62523331
rs795988
rs9518837
rs6585011
rs16980431
rs2721884
rs10119693
rs72819063
rs2291958
rs7998315
rs227442
rs387171
rs838134
All
−0.50
−0.25
0.00
0.25
MR leave−one−out sensitivity analysis for
acteria.p_Bacteroidetes.c_Bacteroidia.o_Bacteroidales.f_Bacteroidaceae.g_Bacteroides.s_Bacteroides_dorei) || id:ebi−a−GCST90027824' on
